# Predicting technostress: The Big Five model of personality and subjective well-being

**DOI:** 10.1371/journal.pone.0313247

**Published:** 2024-11-04

**Authors:** Dámaris Cuadrado, Inmaculada Otero, Alexandra Martínez, Tania París, Silvia Moscoso

**Affiliations:** Department of Political Science and Sociology, University of Santiago de Compostela, Santiago de Compostela, Spain; Shenzhen University, CHINA

## Abstract

The main goal of the current study is to broaden the knowledge on the association between personality, subjective well-being (SWB) and technostress in an academic context. This research specifically examines the prevalence of technostress in a European university sample. It also explores the relationship between technostress and its dimensions with the Big Five model of personality and with SWB and its affective and cognitive components. Finally, the combined predictive validity of the Big Five and SWB on technostress is tested. The sample was composed of 346 undergraduate students. Correlational and multiple regression analyses were carried out. Results show that fatigue and anxiety are the most frequently experienced dimensions of technostress. Emotional stability, openness to experience, and SWB are negatively and significantly correlated to technostress. Multiple regression analyses show that the Big Five factors and SWB account for technostress variance, the main predictor being the affective component of SWB. These results contribute to a more comprehensive understanding of technostress and suggest that personality traits and SWB are important factors in its prediction. The theoretical and practical implications will be discussed.

## Introduction

Steady technological advancement is substantially increasing the use of Information and Communication Technologies (ICTs) in our daily lives. ICTs allow us to be more efficient, accurate, and productive in our activities, to have quick and easy access to information, and to keep in touch with relatives, friends, and coworkers. Despite their benefits, previous studies have shown that ICTs can negatively affect our lives [1–6]. Spending long hours in front of screens, uncontrolled use of ICTs, and a lack of breaks can lead to stress and psychological pressure in users. These symptoms are commonly referred to as technostress.

Technostress affects the lives of individuals in multiple aspects. In the occupational domain, technostress has been linked to employees’ physical and psychological problems, family-work conflict, general and occupational distress and anxiety, work exhaustion, burnout, and lower rates of job performance, work satisfaction, and organizational commitment [3, 5–13]. Similar to the workplace, the academic field has undergone a profound digitalization in recent years. Unfortunately, research in this context has been more limited than in occupational settings [14, 15]. Still, some recent findings indicate that students suffering from technostress show a variety of negative effects. Lower academic performance, higher burnout and anxiety rates, and greater exhaustion levels than their non-technostressed peers can be cited [16–22]. The seriousness of these consequences makes the study of technostress a critical issue for academic institutions.

The validity of individual characteristics to predict technostress has been examined in the recent years, particularly, in the occupational context. Among them, demographic characteristics, cognitive and emotional intelligence, and personality can be cited. Still, personality and subjective well-being have yet to be fully examined regarding technostress in the educational field. For this reason, the main goals of the current research are (1) to examine the prevalence of technostress in a sample of university students; (2) to study its relationship with (a) the dimensions of the Big Five model of personality and with (b) overall SWB and its affective and cognitive components; and (3) to test the joint capacity of the Big Five model and SWB to explain the variance of technostress.

Technostress can be defined as “*the stress experienced by end users in organizations as a result of their use of Information and Communication Technologies (ICTs)*, *caused by an individual’s attempts to deal with constantly evolving ICTs and the changing of physical*, *social*, *and cognitive responses demanded by their use*” [3]. Following the model developed by Llorens and colleagues [23], technostress is a multidimensional construct composed of four dimensions (i.e., anxiety, fatigue, skepticism, and low competence). *Anxiety* occurs when the use of ICTs gives individuals high levels of unpleasant physiological tension and discomfort. *Fatigue* is characterized by mental and cognitive exhaustion derived from the use of ICTs. *Skepticism* refers to negative evaluations made regarding the use of ICTs and is related to attitudes of cynicism and doubtfulness. Finally, *low competence* consists of subjects’ negative thoughts about their own ability to successfully work with ICTs.

Empirical evidence indicates that personality traits explain subjects’ predisposition to suffer from technostress. Self-efficacy and trait/state anxiety are two personality variables that have been the most studied in scientific literature [24–28]. However, the relationship between technostress and its dimensions with other personality characteristics, such as the Big Five model, has not been examined in detail. Additionally, previous results on these relationships are often mixed, ranging from almost-null to moderate correlations [see, for instance, 11,29–31], showing high variability in results, and creating a conflicting picture for researchers and practitioners.

The Big Five model is the most accepted model to explain an individual’s normal personality and it has been shown to predict important occupational and academic criteria [32–39]. This model postulates that an individual’s personality is explained by five dimensions (i.e., emotional stability, extraversion, openness to experience, agreeableness, and conscientiousness).

*Emotional stability* (ES), as opposed to neuroticism, refers to an individual’s ability to control their emotions. People described as emotionally stable are calm, relaxed, and even-tempered, whereas neurotic subjects tend to experience nervousness, anger, guilt, and are more vulnerable to stress and anxiety [40–43]. Since the latter tend to face difficulty in dealing with daily stimuli, it is possible that they experience negative emotions such as fear, hostility, concern, and psychological strain when exposed to ICTs. Failures or interruptions caused by ICTs could be perceived as a source of tension and concern. Although evidence on the ES-technostress relationship is scarce, results in the occupational domain suggest that this dimension is one of the most strongly related to technostress [see, 11]. In the academic context, results by Wang et al. [31] follow the same line.

*Extraversion* (EX) describes individuals who are social, assertive, dominant, and optimistic [40–43]. Since they enjoy interacting with people, the use of ICTs in the academic context could be perceived as a means to satisfy their social needs, allowing them to engage with peers, instructors, and the academic community in general. Previous research has shown that extraverted individuals are genuinely comfortable and confident in computer-mediated interactions [44]. It has been argued that they are motivated to preserve a positive image towards others [45] and, consequently, extraverted subjects could be predisposed to use ICTs as a way to make a favorable impression. As they tend to be socially dominant and are comfortable leading others, ICTs could help them become more prominent in online-teamwork. Empirical results on the EX-technostress relationship are mixed. Some studies in the occupational field show a negative relationship between the variables [e.g., 11,46]. Other studies show a positive association [e.g., 30]. In the educational context, Korzynksi et al. [29] provide a positive but almost null result and Wang et al. [31] report a small negative correlation.

*Openness to experience* (OP) is associated with an active imagination, an interest in values and ideas, and an attention to internal feelings [40–43]. Open individuals are also intellectually curious, oriented towards learning, and they look for opportunities to acquire expertise in new areas [47, 48]. They might perceive ICTs as a means to expand their knowledge and master academic tasks, and, therefore, be less prone to developing negative attitudes. Also, open subjects might feel motivated to solve potential issues with ICTs. Since they are mentally flexible and open to new approaches, they could perceive the use of ICTs as less complicated than those individuals scoring lower in this dimension, resulting, perhaps, in a less stressful experience. Empirical evidence suggests the existence of a negative relationship between the variables. However, the results also show an important variability, ranging from almost null to moderate effect sizes [see, 11,49].

*Agreeableness* (A) describes cooperative, friendly, trustworthy, and tolerant individuals. They reject advantages at the cost of others and are compliant with norms and regulations [40–43]. Thus, highly agreeable students might be more willing to accept the use of ICTs when required by instructors or academic institutions to facilitate collaboration. Likewise, they tend to take pleasure in supporting and accepting help from others, useful qualities when the use of ICTs turns complex or problematic. Furthermore, as they are trusting of others and easier to persuade, they could develop positive attitudes towards ICTs if they are demanded in the academic context. Most of the empirical research has shown negative correlations between A and technostress. However, as seen with other personality dimensions, the variability of results is an important concern. The results reported in primary research vary from virtually zero [e.g., 11,50] to moderate effect sizes [e.g., 31].

The fifth factor, *Conscientiousness* (C), refers to the control of impulses and involves the active process of planning, organizing, and performing tasks. Those scoring high in C are responsible, work-oriented, and self-disciplined [40–43]. As such, it is probable that they are willing to accept ICTs as an efficient instrument to increase their academic performance. Traits like meticulousness and attention to detail can help prevent problems associated with the use of ICTs. Given their inner drive towards achievement, individuals with these traits are expected to be proficient in the use of technology. This could lead to the development of positive perceptions and attitudes towards ICTs. However, empirical evidence on this relationship has also reported mixed results. Some studies inform small positive correlations [e.g., 45], while others, moderate and negative correlations [e.g., 31].

Like the Big Five model of personality, subjective well-being (SWB) is another characteristic that could explain the predisposition of individuals to suffer from technostress. The most widely accepted model of SWB is that of Diener [51–53], which describes SWB as the evaluations individuals make of their lives. SWB consists of a cognitive and an affective component. The first refers to judgments of life satisfaction, and the second refers to the balance between positive and negative emotions experienced by an individual [54–56]. Thus, subjects scoring high in SWB are satisfied with life, and their positive emotions prevail over negative ones. Although SWB has been linked to relevant organizational criteria both in the occupational [e.g., 57–59] and educational domains [60–62], its relationship with technostress is still understudied.

From a theoretical point of view, a negative relationship between technostress and SWB could be expected. According to Salanova and colleagues [63], technostress dimensions can be classified into an affective, an attitudinal, and a cognitive component. The affective component of technostress includes the dimensions of anxiety and fatigue. Comparable to the affective component of SWB, it refers to the emotions that arise when subjects use ICTs. Therefore, we could anticipate that students who tend to experience negative emotions towards life events also manifest negative emotions in specific scenarios, such as when ICTs are used for academic purposes. On the other hand, since the cognitive component of SWB involves evaluating life satisfaction, individuals who are predisposed to be dissatisfied with life may also feel displeased with their abilities. This could result in negative evaluations of their own efficacy when working with ICTs (i.e., technostress-related low competence).

Empirical research examining this relationship (technostress-SWB) has focused particularly on the working population and has shown a predominant interest in evaluating the cognitive component of SWB. Some studies reveal a small to moderate negative relationship between employees’ technostress and life satisfaction [see, for instance, 14,64,65]. The limited number of studies assessing the affective component suggests a link between the variables, especially between negative emotions and technostress [see 64,66]. The results indicate that subjects who present a greater predisposition to feel negative emotions towards life events are also those who are more likely to experience technostress. Lastly, empirical evidence provided by Hang et al. [67] shows a clear negative association between overall SWB and the dimensions of technostress, ranging from small to moderate correlations.

### Aims of the study and hypotheses

The current research has three main objectives. First, to examine the prevalence of technostress in a sample of European university students. Second, to explore the relationship between technostress and its dimensions with (1) the Big Five model of personality and (2) SWB and its components. Third, to examine the validity of the Big Five factors along with SWB to predict technostress. To the best of our knowledge, no prior study has tested the joint effects of these variables in the prediction of this phenomenon in the academic field.

Following the theoretical and empirical rationale presented above, we propose the following hypotheses:

*Hypothesis 1*. The Big Five personality factors, independently, correlate negatively with technostress and its dimensions.
*Hypothesis 2*. Overall SWB correlates negatively with technostress and its dimensions.
*Hypothesis 2a*. The cognitive component of SWB correlates negatively with technostress and its dimensions, meaning that the subjects with a higher life satisfaction are less technostressed.
*Hypothesis 2b*. The affective component of SWB correlates negatively with technostress and its dimensions, meaning that the subjects whose positive emotions prevail over the negative emotions are less technostressed.

## Method

### Participants and procedure

The sample consisted of 346 students who were enrolled in degree programs within the field of social sciences at a Spanish University (112 men and 234 women). The mean age was 21.15 years old (*SD* = 4.63, range = 17–55). Data was gathered in class sessions from April 18^th^ 2023 to May 16^th^ 2023, where the purpose of the study was explained to the students. Those who voluntarily agreed to participate provided informed written consent. Next, an anonymous online questionnaire was administered, and no personal identification was collected. Ethical approval to conduct the study was obtained from the Bioethics Committee of the University of Santiago de Compostela (Code of approval: USC15/2023).

## Materials

### Technostress

Technostress was assessed using the RED-TIC Scale designed by Salanova et al. [68]. The scale consists of 16 items structured in four dimensions: (1) anxiety (e.g., “I feel tense and anxious when working with ICTs”); (2) fatigue (e.g., “When I finish working with ICTs, I feel exhausted”); (3) skepticism (e.g., "I am cynical about the contribution of ICTs to my work"); and (4) low competence (e.g., “I am uncertain about successfully completing tasks when I use ICTs”). Subjects had to indicate the frequency they experienced the situations described in each item during their studies, using a 5-point scale, where 1 = never and 5 = always/every day. The internal consistency coefficients (Cronbach´s alpha) in this study were .83, .88, .75, .80, and .91 for anxiety, fatigue, skepticism, low competence, and the overall scale of technostress, respectively.

**The Big Five model of personality.** The Big Five model of personality was assessed using a Spanish adaptation of Saucier’s Mini-markers scale [69] composed of 35-items. Each personality factor was assessed with 7 adjectives (e.g., emotional, energetic, adventurous, kind, reliable for ES, EX, OP, A, and C, respectively). Subjects had to indicate the extent to which they feel represented by each adjective using a 5-point scale, where 1 = not at all and 5 = a lot. Previous studies have examined the psychometric properties of this personality inventory. For instance, Tavares [70] found that the instrument is a reliable measure and that its factorial structure fits the five dimensions of the model. The internal consistency coefficients (Cronbach´s alpha) were .83, .66, .74, .72, and .77, for EX, ES, OP, A, and C, respectively.

#### Subjective well-being

The cognitive component of SWB was assessed using the 5-item Scale of Satisfaction with Life [71]. The items (e.g., “I am completely satisfied with my life”) were answered using a 5-point scale (from 1 = totally disagree to 5 = totally agree). The internal consistency reliability (Cronbach´s alpha) of the scale was .83 in this study. The affective component of SWB was assessed with the Scale of Positive and Negative Affective Experience, SPANE [55, 56], which describes feelings and emotions using 14 adjectives. Seven adjectives were positive (e.g., happy) and seven adjectives were negative (e.g., stressful). Subjects had to indicate the frequency with which they experience each emotion using a 5-point scale (from 1 = never to 5 = always). The SPANE Scale provides three scores: (1) a positive affect score; (2) a negative affect score; and (3) an emotional balance score (i.e., the affective component of SWB). The balance was calculated by subtracting the negative affect score from the positive affect score. The internal consistency coefficients (Cronbach´s alpha) of the positive affect, negative affect, and emotional balance were .87, .82, and .89, respectively, for the current sample. Last, we estimated a measure of overall SWB by creating a composite of the cognitive and the affective components. The internal consistency using Mosier’s estimation of composites reliability was .91.

## Results

### Descriptive statistics of technostress

Table 1 presents the descriptive statistics both for the overall measure of technostress and its dimensions. The average score could range from 1 to 5. Although the mean values do not exceed the midpoint of the scale (i.e., 3 points), some dimensions appear to be more prevalent than others. In this case, fatigue is the most frequently experienced dimension of technostress (*M* = 2.90, *SD* = 1.05), followed by anxiety (*M* = 2.38, *SD* = 1.01). The lowest average score was for skepticism (*M* = 1.92, *SD* = 0.77).

**Table 1:** Descriptive statistics of the technostress measure.

	Items	*M*	*SD*	Max.	Min.
**Technostress**	16	2.31	0.75	5	1
Anxiety	4	2.38	1.01	5	1
Fatigue	4	2.90	1.05	5	1
Skepticism	4	1.92	0.77	5	1
Low competence	4	2.01	0.82	5	1

*Note*. *N* = 346; *M* = mean, *SD* = standard deviation; Max. = maximum individual mean score; Min. = minimum individual mean score.

Table 2 shows the frequency (expressed in percentage) with which the individuals admitted to having experienced, on average, technostress and its dimensions.

**Table 2:** Percentage of students engaging in technostress at each level of frequency.

	Technostress	Anxiety	Fatigue	Skepticism	Low competence
**Never**	33.24	30.71	16.40	44.94	40.89
**Hardly ever**	28.45	29.26	24.35	28.76	31.43
**Sometimes**	18.84	17.99	23.27	17.56	16.55
**Usually**	13.60	14.96	24.35	7.15	7.95
**Always**	5.87	7.08	11.63	1.59	3.18

*Note*. *N* = 346.

Results show that 66.55% of the sample reported to having experienced one or more indicators of technostress at least once during their studies. Following the line of the results presented in Table 1, fatigue is the dimension occurring with the highest frequency. Of the students who took part in the study, only 16.40% admitted to never having experienced any signs of ICTs-related fatigue, while 11.63% indicated suffering from it every time they work with technology. Skepticism appears to be the least frequently experienced dimension, with 44.94% of the sample reporting never having been skeptical of the use and value of ICTs.

#### Bivariate correlations

Tables 3 and 4 report the bivariate correlations among technostress and its dimensions with the Big Five model of personality and with SWB and its affective and cognitive components. Specifically, Table 3 reports the observed correlations among the variables and Table 4 reports the true correlations for the key relationships in this research. The true correlations were corrected for measurement error in the independent and the dependent variables. We describe the results reported in Table 4 in the following paragraphs.

**Table 3:** Observed correlations among the variables.

	*M*	*SD*	1	2	3	4	5	6	7	8	9	10	11	12	13	14	15	16	17
** 1. Sex**	-	-	-																
** 2. Age**	*21*.*15*	*4*.*63*	-.12*	-															
** 3. Technostress**	*2*.*31*	*0*.*75*	.17**	-.13*	*(*.*91)*														
** 4. Anxiety**	*2*.*38*	*1*.*01*	.16**	-.10	.88**	*(*.*83)*													
** 5. Fatigue**	*2*.*90*	*1*.*05*	.23**	-.12*	.81**	.60**	*(*.*88)*												
** 6. Skepticism**	*1*.*92*	*0*.*77*	.03	-.07	.75**	.53**	.48**	*(*.*75)*											
** 7. Low competence**	*2*.*01*	*0*.*82*	.09	-.15**	.82**	.72**	.49**	.52**	*(*.*80)*										
** 8. ES**	*3*.*37*	*0*.*58*	-.27**	.08	-.28**	-.28**	-.26**	-.14**	-.22**	*(*.*66)*									
** 9. EX**	*3*.*42*	*0*.*75*	-.09	.19**	-.03	.00	.03	-.07	-.08	.18**	*(*.*83)*								
**10. OP**	*3*.*55*	*0*.*62*	-.22**	.11*	-.18**	-.10	-.15**	-.16**	-.21**	.28**	.40**	*(*.*74)*							
**11. A**	*4*.*05*	*0*.*52*	-.03	.06	-.09	-.11	.03	-.13*	-.12*	.37**	46**	37**	*(*.*72)*						
**12. C**	*3*.*62*	*0*.*62*	.20**	.09	-.09	-.05	-.04	-.10	-.12*	.26**	23**	45**	36**	*(*.*77)*					
**13. SWB**	-	-	-.11*	.19**	-.31**	-.33**	-.18**	-.20**	-.28**	50**	38**	25**	37**	28**	*(*.*91)*				
**14. Life satisfaction**	*3*.*17*	*0*.*84*	.01	.14**	-.16**	-.17**	-.07	-.14**	-.16**	34**	36**	22**	34**	28****	-	*(*.*83)*			
**15. Emotional balance**	*0*.*65*	*1*.*36*	-.20**	.19**	-.37**	-.40**	-.25**	-.21**	-.33**	53**	30**	21**	31**	20**	-	51**	*(*.*89)*		
**16. Positive affect**	*3*.*36*	*0*.*74*	-.13*	.16**	-.30**	-.34**	-.17**	-.21**	-.28**	.44*	37**	28**	40**	29**	-	52**	88**	*(*.*87)*	
**17. Negative affect**	*2*.*70*	*0*.*79*	.23**	-.18**	.36**	.38**	.28**	.17**	.31**	-.49**	-.17**	-.10	-.16**	-.07	-	-.39**	-.90**	-.58**	*(*.*82)*

*Note*. *N* = 346; Reliability coefficients are presented in the diagonal; Sex was coded 0 for male and 1 for female; ES = emotional stability; EX = extraversion; OP = openness to experience; A = agreeableness; C = conscientiousness; SWB = subjective well-being.

*p < .05

**p < .01.

**Table 4:** Corrected correlations (ρ) among the variables.

	Technostress	Anxiety	Fatigue	Skepticism	Low competence
**Emotional stability**	-.36**	-.38**	-.34**	-.20**	-.30**
**Extraversion**	-.03	.00	.04	-.09	-.10
**Openness to experience**	-.22**	-.13*	-.19**	-.21**	-.27**
**Agreeableness**	-.11	-.14**	.04	-.18**	-.16**
**Conscientiousness**	-.11	-.06	-.05	-.13**	-.15**
**SWB**	-.34**	-.38**	-.20**	-.24**	-.33**
**Life satisfaction**	-.18**	-.20**	-.08	-.18**	-.20**
**Emotional balance**	-.41**	-.47**	-.28**	-.26**	-.39**
**Positive affect**	-.34**	-.40**	-.19**	-.26**	-.34**
**Negative affect**	.42**	.46**	.33**	-.22**	.38**

*Note*. *N* = 346; SWB *=* subjective well-being.

*p < .05

**p < .01.

Starting with the personality variables, the results show that emotional stability and openness to experience are valid predictors of general technostress. The corrected correlation for emotional stability is ρ = -.36. The result for openness to experience is ρ = -.22. Conscientiousness and agreeableness show negative but non-significant results. In the case of extraversion, the result is essentially zero. In regard to the specific dimensions of technostress, emotional stability and openness to experience appear to be valid predictors of the four dimensions. The correlations are negative and significant in all instances, ranging from ρ = -.38 for anxiety to ρ = -.20 for skepticism in the case of emotional stability, and from ρ = -.27 for low competence to ρ = -.13 for anxiety in the case of openness to experience. Conscientiousness and agreeableness are valid predictors of certain dimensions. These are skepticism (ρ = -.13 and ρ = -.18, respectively) and low competence (ρ = -.15 and ρ = -.16, respectively). Agreeableness is also a valid predictor of anxiety (ρ = -.14). Finally, no significant correlations were found between extraversion and the four dimensions of technostress for this sample (ρ ranging from -.10 to .04). Overall, the results indicate that emotional stability is the strongest personality characteristic in the prediction of general technostress and of the specific dimensions of anxiety, fatigue, and low competence. The best predictor of skepticism is openness to experience. These results partially support Hypothesis 1, as emotional stability and openness to experience significantly and negatively correlate with technostress and its dimensions. However, agreeableness and conscientiousness are only valid predictors of some dimensions, and extraversion did not predict any criteria.

In regard to SWB, the results show that overall SWB, its affective component (i.e., emotional balance), and, partially, its cognitive component (i.e., life satisfaction), negatively and significantly correlate with technostress and its dimensions. The best predictor of general technostress is emotional balance with a corrected correlation of ρ = -.41. Life satisfaction shows a lower but also significant result of ρ = -.18, and overall SWB of ρ = -.34. In the prediction of the specific technostress’ dimensions, the results for overall SWB are negative and significant in all the cases, ranging from ρ = -.38 for anxiety to ρ = -.20 for fatigue. Both life satisfaction and emotional balance are valid predictors of skepticism (ρ = -.18 and ρ = -.26, respectively), anxiety (ρ = -.20 and ρ = -.47, respectively), and low competence (ρ = -.20 and ρ = -.39, respectively). In the case of fatigue, emotional balance is the only valid predictor (ρ = -.28). These results indicate that emotional balance is a stronger predictor of general technostress and its dimensions than life satisfaction, suggesting the superiority of the affective component of SWB over the cognitive component in the prediction of this phenomenon. Overall, the obtained results support Hypothesis 2 and Hypothesis 2b. Hypothesis 2a is only partially supported as life satisfaction is not a valid predictor of fatigue.

### Multiple regression analyses

Table 5 shows the results of the multiple regression analyses. The correlations corrected for measurement error in the dependent and the independent variables were used to conduct the analyses, because the presence of artifactual errors violates the independence-of-errors assumption and, consequently, biases the obtained parameters [72–75]. Likewise, we estimated the squared population cross-validity coefficient (*R*^2^_cv_) using Browne´s formula [76] in order to control the biases on *R*, *R*^2^, and adjusted *R*^2^ due to capitalization on chance. This phenomenon produces a systematic attenuation of *R*^2^ when the regression coefficients obtained for a specific sample are applied to a different sample.

**Table 5:** Regression analyses of the Big Five model of personality and SWB on technostress.

	β	*R*	*R* ^2^	*R* ^2^_adj_	*R* ^2^_cv_
**Personality**		.403	.162	.150	.140
Emotional stability	-.389**				
Extraversion	.072				
Openness to experience	-.212**				
Agreeableness	.129				
Conscientiousness	.074				
**LS + EB**		.417	.174	.169	.167
Life satisfaction	.095				
Emotional balance	-.466**				
**Personality + LS + EB**		.483	.233	.217	.204
Emotional stability	-.105				
Extraversion	.189**				
Openness to experience	-.265**				
Agreeableness	.069				
Conscientiousness	.081				
Life satisfaction	.042				
Emotional balance	-.406**				
Δ *LS+EB over personality*			.071		

*Note*. *N* = 346; β = standardized regression weight; *R* = multiple correlation; *R*^2^ = explained variance; *R*^2^_adj_ = adjusted *R*^2^; *R*^2^_cv_ = squared population cross-validity coefficient; LS = life satisfaction; EB = emotional balance; Δ = increment in *R*^2^.

* *p* < .05

** *p* < .01

The first part of the analyses was carried out to test the predictive validity of the Big Five model of personality. When the five dimensions of personality are entered together in the regression analysis, the results show a multiple correlation of *R* = .403 and a squared multiple correlation of *R*^2^ = .162, meaning that the Big Five model of personality contributes to the explanation of 16.2% of the technostress variance. In alignment with the correlational results presented above, the best predictor of technostress is emotional stability, with a beta coefficient of β = -.389, followed by openness to experience with a result of β = -.212. The beta coefficients for the remaining dimensions are not statistically significant.

When the joint predictive validity of the cognitive and affective components of SWB is tested, the results show a multiple correlation of *R* = .417 and a squared multiple correlation of *R*^2^ = .174. Thus, life satisfaction and emotional balance explain 17.4% of the technostress variance. The beta coefficients indicate that emotional balance is the main predictor of technostress with a beta value of β = -.466. The result for life satisfaction is a non-significant beta weight of β = .095.

The third regression analysis shows that, when the Big Five personality dimensions are entered in the regression equation together with the SWB components, the explained variance of technostress increases to 23.3% (*R*^2^ = .233 and *R* = .483). We also tested the significance of the difference between this multiple correlation (*R* = .483) and the multiple correlation obtained in the first regression analysis (*R* = .403) when only the Big Five dimensions were entered in the equation. For this, we used the formula provided by Guilford [77] for the estimation of the statistical significance of the difference between multiple correlations calculated with a different number of independent variables. The result was an *F* value of 15.62 (*p* < .01), meaning that the estimations are distinct with a probability greater than 99%. The increment produced in *R*^2^ (Δ) by the SWB components over the personality dimensions is *R*^2^ = .071 (7.1% of explained variance).

In regard to the specific predictive weights, both the affective and the cognitive components of SWB show similar results to the previous regression analysis, emotional balance being the key variable in the prediction of technostress with a beta value of β = -.406. In the case of the Big Five model, the beta values of two personality dimensions show substantial alterations when compared to the results produced in the previous analyses. First, the predictive validity of emotional stability decreases from β = -.389 (*p* < .01) to β = -.105 (*p* > .05). As emotional stability and emotional balance share a substantial portion of their variance (*r* = .53, that increases to ρ = .69 when measurement error is corrected), the regression analysis assigns the shared explanatory capacity of technostress to the affective component of SWB. Consequently, this lowers the beta weight of emotional stability. Second, the beta value of extraversion increases from β = .072 (*p* > .05) to β = .189 (*p* < .01). In this case, the existence of a potential suppressor effect must be examined. With this purpose, a regression analysis from which both components of SWB were eliminated one by one from the equation to test the variations on the magnitude of extraversion beta weight and *R*^2^ was carried out. The results are presented in Table 6.

**Table 6:** Analysis of a suppressor effect on extraversion.

	β	*R*	*R* ^2^
**Step 1.**		.430	.185
Extraversion	.115*		
Life satisfaction	.055		
Emotional balance	-.483**		
**Step 2.**		.428	.183
Extraversion	.129*		
Emotional balance	-.455**		
**Step 3.**		.188	.035
Extraversion	.058		
Life satisfaction	-.205**		

*Note*. *N* = 346; β = standardized regression weight; *R* = multiple correlation; *R*^2^ = explained variance.

* *p* < .05

** *p* < .01

As can be seen, when the cognitive component of SWB (i.e., life satisfaction) is removed from the equation, the change produced on the beta weight of extraversion and *R*^2^ is minimal. However, when the affective component (i.e., emotional balance) is eliminated, the beta weight of extraversion drops to half of its magnitude (from β = .115 to β = .058), and the explained variance changes from *R*^2^ = .185 to an almost null result (*R*^2^ = .035). These results support the presence of a suppressor effect. According to MacKinnon [75, 78], when a suppressor effect is detected, its significance needs to be tested. For this, the Sobel’s test was calculated using the software created by Preacher and Leonardelli [79]. The 95% confidence interval of the suppressor effect was also estimated using the distribution of the product of the regression coefficients (*z* test). For this, the software developed by Tofighi and Mackinnon [80] was used. The results are shown in Table 7. As can be seen, the suppressor effect is significant. The Sobel’s test is 2.36 (*p* = .019) and the 95% confidence interval ranges from .009 to .093.

**Table 7:** Analysis of the significance of the suppressor effect.

	β	*R* ^*2*^	Sobel test	αβ (*z* test)	95% CI αβ	
					LL	UP
**Effects on EX w/o EB**	.058	.035	2.35 (*p* = .019)	.046	.009	.093

*Note*. EX = extraversion; EB = emotional balance; β = new predictive weight of EX after removing from the regression equation EB; *R*^*2*^ = explained variance of technostress; αβ (test *z*) = level of significance for the confidence interval; 95% CI αβ LL / UL = lower limit and upper limit of the 95% confidence interval of the suppressor effect.

## Discussion

This research aimed to expand the knowledge of technostress and its relationship with individual characteristics in the academic domain. Specifically, its three main objectives were to examine the prevalence of technostress in a sample of European university students, to explore the relationship between technostress and its dimensions with (1) the Big Five model of personality and (2) SWB and its components, and, lastly, to examine the validity of the Big Five factors along with SWB to predict technostress.

This research has contributed to the study of technostress in several ways. The first contribution has been to show that fatigue and anxiety are the most common forms of technostress experienced among students. Despite the integration of ICTs into student’s daily lives, results suggest that they can still experience mental exhaustion and discomfort from their use. This finding is noteworthy given that most participants in the study are digital natives (93.9% were 25 years old or younger when the study was carried out) and are familiar with and continuously exposed to ICTs [81, 82].

The second contribution has been to show that emotional stability and openness to experience are key personality factors in accounting for technostress variance. Emotional stability appeared to be the strongest predictor within the Big Five model, supporting the idea that individuals with less control of their emotions are more susceptible to suffer from this phenomenon. This finding is also consistent with prior research in the academic domain, such as Wang et al. [31] and Korzynski et al. [29], who found a negative correlation between a specific trait of emotional stability (i.e., self-esteem) and technostress. On the other hand, openness to experience also predicted technostress, suggesting that individuals who are intellectually curious and oriented towards problem solving may be less technostressed. Although empirical evidence with working samples on this relationship is inconclusive [see, e.g., 11,49], results obtained in the educational field are similar to those of the current study [see, 31].

The third contribution of this study has been to show that students with higher levels of SWB, particularly in its affective component, tend to experience lower levels of technostress. This aligns with the findings produced for emotional stability and suggests that emotional regulation is crucial in predicting technostress. Experiencing positive emotions while minimizing negative reactions to life events could mitigate psychosocial risks in academic settings. Previous studies involving working and general populations also supported this association. For instance, Candel [64] emphasized the dominance of the affective component in the prediction of a similar phenomenon (i.e., techno-wellness).

The fourth contribution has been to test a comprehensive predictive model of technostress that is, to the best of our knowledge, the first of its kind in scientific literature. The results support the complementary use of emotional stability and openness to experience, together with SWB (especially its affective component) in the prediction of technostress. Jointly, these two personality factors and SWB explained 23.3% of technostress variance. Also, these results reveal a suppressor effect. The presence of the affective component made extraversion a significant predictor of technostress. This effect has not been previously identified in scientific literature. Consequently, this is the fifth, and final, contribution of this research.

In conclusion, this research helped expand the current understanding of technostress by exploring its prevalence and addressing how individual characteristics can contribute to the experience of this phenomenon among university students.

### Implications for research and practice

#### Theoretical implications

From a theoretical point of view, our results have some implications for researchers on technostress. First, this study broadens the knowledge on the nomological network of this construct and expands previous research on its correlates. Our results showed that individual characteristics share variance with technostress, providing a more robust theoretical comprehension of this phenomenon.

Second, prior studies examining the relationship between technostress and individual characteristics show a high variability in their results [e.g., 11,29–31]. An additional gap in previous research is the absence of corrected data, which could also increase the variability of the results. In our study we perform artifactual corrections of the correlations which is crucial for clarifying the associations between the variables. This ultimately contributes to a more comprehensive body of quantitative data that can be used in future meta-analyses on the topic in order to estimate the true magnitude of the effect sizes and to test whether the observed variability is real or is due to artifacts [72, 83].

Third, previous research on technostress has primarily focused on working samples from North America and Asia. By exploring this phenomenon and its correlations within the higher education context in Europe, researchers will be able to test the generalization of results across different regions and examine potential moderating variables, such as cultural differences or academic levels.

Last, the current research reveals a novel suppressor effect. Suppressor effects involving SWB have been previously discussed [see, for example, 84], however the effect found in our analyses had not been previously identified in scientific literature. As described, the presence of the affective component of SWB made extraversion a significant predictor of technostress. This finding opens new directions for researchers to explore.

#### Practical implications

This research offers valuable insights for academic administrators, instructors, and ICTs users in educational settings. First, our results can guide in the design of initiatives to mitigate technostress. It was shown that certain dimensions of technostress are more prevalent than others, even among individuals considered digital natives. Understanding which specific manifestations are most commonly experienced can help prioritize interventions effectively.

Second, our results support the importance of preventive measures in higher education. Academic administrators should invest both in digitalization and in the prevention of ICTs´ psychosocial risks. Practical initiatives, such as education about time-management in the usage of ICTs, promoting digital disconnection, and emphasizing ergonomics, could be implemented.

Third, higher education instructors are encouraged to participate in prevention initiatives. As evidence indicates that ICTs-related work overload can develop into high levels of fatigue [85, 86], coordinating with colleagues could help prevent excessive digital workloads assigned to students.

Fourth, empirical evidence suggests that emotional stability and openness to experience exhibit strong resistance to change from young adulthood onwards [87–89]. Therefore, rather than modifying these characteristics, academic administrators should focus their assessment on identifying the target populations for interventions.

Fifth, the affective component of SWB appeared to be a valid predictor of technostress. In contrast to personality traits and their resistance to change, the affective component of SWB is a more suitable variable to perform modulation interventions. In this line, meta-analytic research [e.g., 90] highlights the role of instructors’ social support (i.e., affective support, appraisal, informative support, instrumental support) in enhancing positive emotions and reducing negative emotions in academic settings. In this regard, it is crucial to train instructors to provide positive feedback, communicate academic expectations, manage resources, and foster student motivation. Furthermore, instructors’ behavioral tendency to be empathic and warm towards others should be considered in selection procedures.

Lastly, student participation is essential in institutional efforts to prevent and mitigate technostress. This proactive approach not only supports the individual’s own well-being but makes the students responsible in the promotion of good practices in the use of ICTs.

#### Limitations of the study

Last, it is important to consider that this study has some limitations. First, the sample size is limited and, accordingly, sampling error might cause random variations in the validity coefficients. Since sampling error is a systematic error, it cannot be controlled in primary research unless a quantitative accumulation of results (i.e., a meta-analysis) is carried out.

Second, the Big Five model of personality was measured using a single-stimulus (SS) instrument. Although the current research was anonymous, it is known that this answer format could be easy to respond to in a socially desirable manner. A solution to this problem is to use quasi-ipsative forced-choice (FC) inventories, as they can control the effects of faking [see, 91,92]. In this sense, it would be worthwhile to examine whether these results would be replicated with quasi-ipsative FC measures in future research.
